# Interaction between Penaeid Shrimp and Fish Populations in the Gulf of Mexico: Importance of Shrimp as Forage Species

**DOI:** 10.1371/journal.pone.0166479

**Published:** 2016-11-10

**Authors:** Masami Fujiwara, Can Zhou, Chelsea Acres, Fernando Martinez-Andrade

**Affiliations:** 1 Department of Wildlife and Fisheries Sciences, Texas A&M University, College Station, Texas, United States of America; 2 East China Sea Fisheries Research Institute, Chinese Academy of Fisheries Sciences, Shanghai, China; 3 Department of Fish and Wildlife Conservation, Virginia Polytechnic Institute and State University, Blacksburg, Virginia, United States of America; 4 Coastal Fisheries Division, Texas Parks and Wildlife Department, Corpus Christi, Texas, United States of America; National Taiwan University, TAIWAN

## Abstract

This study investigated the contribution of shrimp stocks in supporting the production of valuable predator species. Fishery-independent data on white shrimp, brown shrimp, and selected fish species (spotted seatrout, red drum, and southern flounder) were collected from 1986 to 2014 by the Texas Parks and Wildlife Department, and converted to catch-per-unit effort (CPUE). Here, the associations between the CPUEs of fish species as predators and those of shrimp species as prey in each sampled bay and sampling season were analyzed using co-integration analysis and Partial Least Squares Regression (PLSR). Co-integration analysis revealed significant associations between 31 of 70 possible fish/shrimp pairings. The analysis also revealed discernible seasonal and spatial patterns. White shrimp in August and brown shrimp in May were associated with fish CPUEs in bays located along the lower coast of Texas, whereas white shrimp in November was more strongly associated with fish CPUEs in bays located on the upper coast. This suggests the possible influence of latitudinal environmental gradient. The results of the PLSR, on the other hand, were not conclusive. This may reflect the high statistical error rates inherent to the analysis of short non-stationary time series. Co-integration is a robust method when analyzing non-stationary time series, and a majority of time series in this study was non-stationary. Based on our co-integration results, we conclude that the CPUE data show significant associations between shrimp abundance and the three predator fish species in the tested regions.

## Introduction

The shrimp industry is the most valuable fishery in the Gulf of Mexico; it is worth $588 million USD and accounts for 65% (by weight) of the total US shrimp landings [1]. However, the important ecological contribution of shrimp stocks in supporting the production of valuable predator species is underestimated. Recent stock assessment models [2, 3] estimated that approximately 62 billion brown shrimp (*Farfantepenaeus aztecus*) and 14 billion white shrimp (*Litopenaeus setiferus*) are recruited as sub-adults in the Gulf of Mexico each year. The pre-recruitment natural mortality rate (i.e., that of post-larval and juvenile shrimp) was estimated to be 23–61% when predation was included, but only 3% in the absence of predation [4]. This suggests that a significant number of juveniles are preyed upon before becoming available to the fishery, and highlights the potential importance of shrimp as a forage species.

In fisheries, species are considered forage species when they play a role as necessary prey for larger predators, such as larger fish, marine mammals, and seabirds [5]. The term “forage species” has meaning beyond something that is eaten by predators. Its depletion must have a detrimental effect on the predators, and the depletion of forage species due to fisheries is a serious ecological concern because they compete with the needs of these predators. Small- to medium-sized pelagic fishes, such as anchovy (Family Engraulidae), menhaden (*Brevoortia spp*.), and mackerel (*Scomber spp*.), are considered forage species. Shrimp have some characteristics in common with these pelagic species, including: relatively short life spans and high vulnerability to environmental factors, which can obscure the relationship between stock abundance and recruitment [6, 7]; and high, often localized abundances (pelagic fish form schools, while juvenile shrimp aggregate in coastal areas), which can make them more vulnerable to predation.

Major predators of juvenile penaeid shrimp in Galveston Bay, TX, include southern flounder (*Paralichthys lethostigma*), spotted seatrout (*Cynoscion nebulosus*), and red drum (*Sciaenops ocellatus*) [4]. These species support important recreational and commercial fisheries throughout the Gulf of Mexico [8, 9], and are highly sought after by recreational anglers in Texas [10]. The availability of different species of juvenile penaeid shrimp varies seasonally in near- and inshore areas, where high predation occurs. White shrimp are more abundant during late summer and fall [11], whereas brown shrimp are more abundant during the spring and early summer [12]. Consistent with this, analyses of the stomach contents of red drum revealed large numbers of white shrimp in juvenile/adult fish during the fall, but large numbers of brown shrimp, along with menhaden, during spring [13–15]. Southern flounder [4, 16] and spotted seatrout [17, 18] are also known to feed extensively on penaeid shrimp.

As a part of a project aimed at evaluating the importance of penaeid shrimp as a forage species, we investigated the statistical association between time series (1986–2014) data on the catch-per-unit effort (CPUE) of shrimp (white shrimp and brown shrimp) and fish (spotted seatrout, red drum, and southern flounder). Both white shrimp and brown shrimp exhibit an annual life history and utilize coastal marshes and estuaries during their juvenile stages, which are preyed upon by the juvenile and young adult stages of the studied fishes. The data used in this study targeted these stages of shrimp and fishes, and samples were collected in the major bays along the Texas coast as a part of a long-term monitoring program that was independent of any fishery. As co-linearity and non-stationarity are frequent problems in statistical associations of time series data, we used partial least squares regression (PLSR) [19] and co-integration analyses [20], and compared the results.

## Methods

### Data

**Fig 1:**
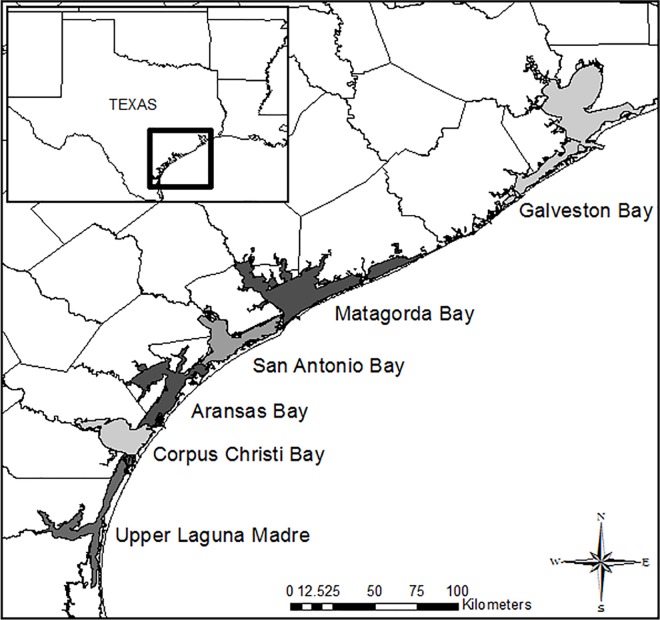
Map showing six bays for monitoring.

Fishery-independent data were collected by the Coastal Fisheries Division of the Texas Parks and Wildlife Department as part of the Marine Resource Monitoring Program conducted in major coastal bays of Texas. In the present study, we analyzed data obtained from Galveston Bay, Matagorda Bay, San Antonio Bay, Aransas Bay, Corpus Christi Bay, and the upper Laguna Madre (Fig 1) from January 1986 to July 2014. The surveys used gill nets and trawls to capture fish and shrimp, respectively (S1 Table).

For fish, gill net sampling was conducted twice a year, for the 10 weeks following the first full week of April (spring sampling) and the 10 weeks following the first full week of September (fall sampling). A total of 45 gill nets were set in each bay system per season, with no less than three gill nets set each week. The gill nets covered the water column from the seafloor to 1.2 meters above the bottom, had a total length of 182.9 m, and were constructed of four continuous 45.7 m-long panels of stretched mesh monofilament webbing of 152 mm, 127 mm, 102 mm, and 76 mm in size. Nets were set perpendicular to the shore, with the smallest mesh (76 mm) nearest the shore; they were deployed around sunset and retrieved around sunrise each day. Organisms greater than 5 mm in total length were identified to the lowest taxonomic level [21]. Data were converted to CPUE (number of individuals caught per hour). For the present study, we selected the CPUEs of spotted seatrout, red drum, and southern flounder, as these are three major targets of recreational fisheries in Texas and the Gulf of Mexico.

For shrimp, bay trawl surveys were conducted monthly. To ensure proper spatial representation, larger bays (Galveston, West Matagorda, San Antonio, Aransas, and Corpus Christi) were stratified into approximately equal-sized upper and lower areas. A total of 20 trawls were conducted in each bay system per month, and scheduled to ensure the temporal representation of samples within a month. The first set of samples was collected during the first half of the month, and the remaining samples were collected during the second half of the month. Sampling was conducted during daylight hours from 30 minutes before sunset to 30 minutes after sunset. The utilized otter trawls had 6.1-m openings and were made of 38-mm stretched nylon multifilament mesh. They were towed for 10 minutes at 3 mph in a semi-circular manner. As with the gill net sampling, captured organisms greater than 5 mm in total length were identified to the lowest taxonomic level [21]. Brown shrimp and white shrimp data were converted to CPUEs (number of individuals caught per unit time).

**Fig 2:**
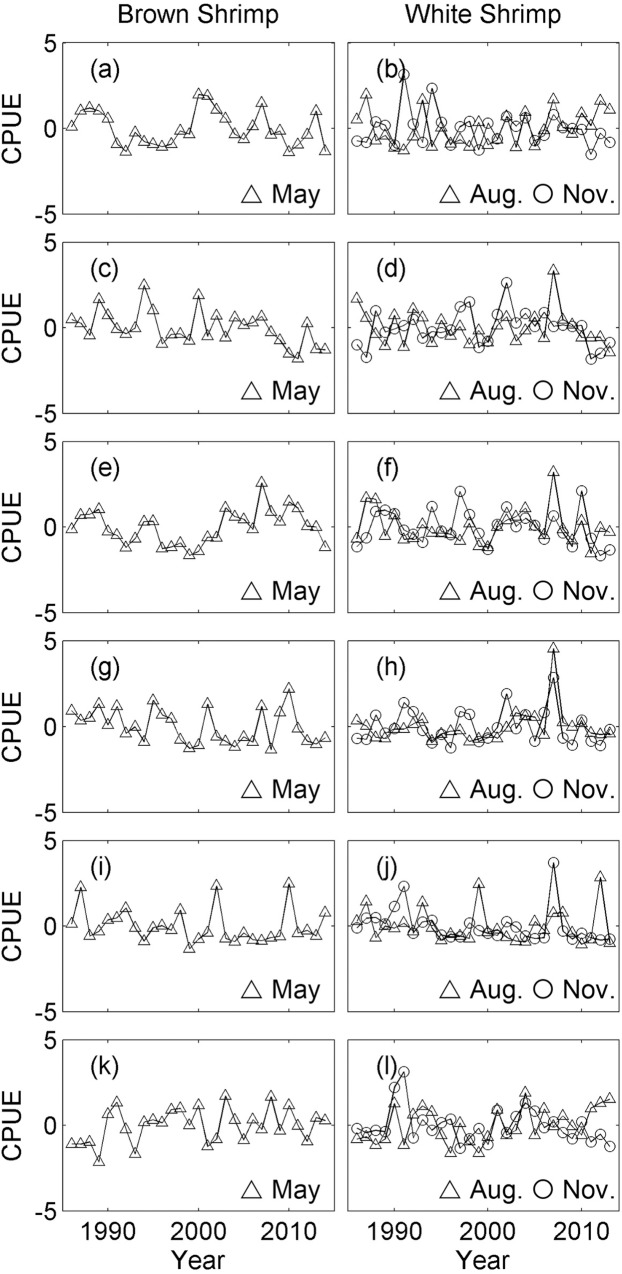
Standardized catch per unit effort (CPUE) of brown shrimp and white shrimp. CPUEs of brown shrimp in May (panels a, c, e, g, i, and k) and white shrimp (panels b, d, f, h, j, and l) in August (circle) and November (triangle). Locations: (a-b) Galveston Bay, (c-d) Matagorda Bay, (e-f) San Antonio Bay, (g-h) Aransas Bay, (i-j) Corpus Christi Bay, and (k-l) Upper Laguna Madre. The original CPUEs were transformed by taking the square root and then standardized by taking the Z-score.

**Fig 3:**
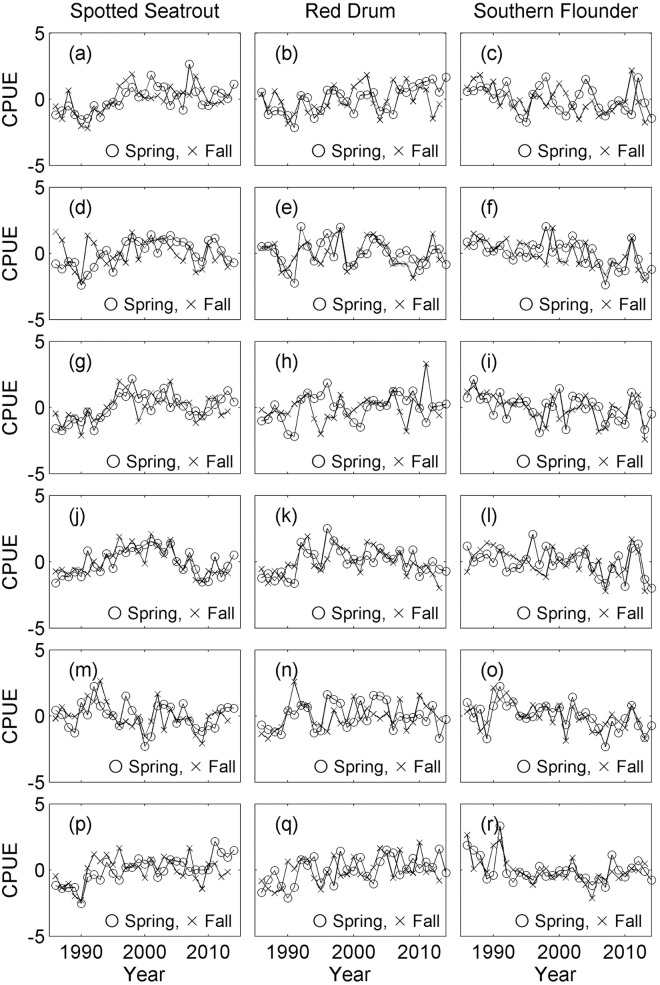
Standardized catch per unit effort (CPUE) of fishes. Left column: spotted seatrout; middle column: red drum; right column: southern flounder. Circles indicate spring, and x indicate fall. Locations: (a-c) Galveston Bay, (d-f) Matagorda Bay, (f-i) San Antonio Bay, (j-l) Aransas Bay, (m-o) Corpus Christi Bay, and (p-r) Upper Laguna Madre. The original CPUEs were transformed by taking the square root and then standardized by taking the Z-score.

For most locations and years, the seasonal peak of brown shrimp was May and those for white shrimp were August and November. Therefore, the CPUEs of shrimp species from those months were used for the analysis (Fig 2). The CPUEs of the three fish species during spring (Fig 3) were compared with the CPUEs of white shrimp in the fall and brown shrimp in the spring of the previous year, while the CPUEs of fish during fall (Fig 3) were compared with CPUEs of white shrimp in the fall and brown shrimp in the spring of the same year. A comparison of fish data in fall and shrimp data from the previous fall would have been another logical choice, but it was not included because it would have decreased the length of the fish data by one year, thereby reducing the power of the analysis. The data from each bay in each season were analyzed separately. All data were transformed by taking the square root to stabilize the variance.

### Partial Least Squares Regression (PLSR) Analysis

Associations between the CPUEs of each fish species and those of each shrimp species for each bay in each fish-sampling season were analyzed using PLSR. This analysis is similar to canonical correlation analysis except that the error term in the PLSR analysis is a univariate, so the analysis assumes the direction of dependency for the variables. As this study aimed to investigate the importance of shrimp as forage species for larger fish species, the fish time series were treated as dependent variables while the shrimp time series were treated as independent variables (Table 1). Similar to canonical correlation analysis, this strategy takes advantage of co-linearity among time series to find components of dependent and independent variables that are associated with one another. The data were standardized by taking z-scores, and then analyzed using the “plsregress.m” function in MATLAB [22]. The significance of each association was determined using a one-at-a-time cross validation. When adding one or more independent components significantly improved the sum of the squared prediction error for the dependent component, we concluded that there was significant association between the dependent and independent variables.

**Table 1:** Ratio between the sum of the squared prediction error without an independent variable and that with an independent variable, as obtained using PLSR analysis. A ratio > 1 indicates a significant association (emphasized with bold and asterisk *) between the fish and shrimp time series (brown and white shrimp combined).

Location	Spring Fish Data			Fall Fish Data		
	Spotted Seatrout	Red Drum	Southern Flounder	Spotted Seatrout	Red Drum	Southern Flounder
Galveston	0.93	0.84	0.79	0.73	0.85	0.91
Matagorda	1.03*	0.89	0.87	0.86	0.95	0.74
San Antonio	0.96	0.85	1.02	0.92	0.89	0.73
Aransas	0.91	0.80	0.93	0.88	1.15*	0.95
Corpus Christi	0.77	1.18*	1.01*	1.12*	0.89	0.97
Upper Laguna Madre	1.22*	0.94	0.74	0.80	1.14*	0.74

### Co-integration Analysis

Co-integration analysis [20] was used to assess the association between two non-stationary time series, which was seen in the majority of the studied time series. Whereas non-stationarity is known to cause spurious results in linear regression, PLSR, and related analyses, co-integration analysis takes advantage of non-stationarity and finds a linear combination of time series that is stationary. The time series involved in such a combination are considered to be co-integrated with one another. Therefore, while PLSR analysis is advantageous in detecting association at high frequency fluctuation when time series are stationary, co-integration analysis is advantageous in detecting association at low frequency fluctuation when time series are non-stationary. Co-integration analysis can be viewed as a type of factor analysis in which two or more time series that can eliminate a non-stationary pattern together are identified. Suppose two non-stationary time series $x_{t}^{\left(1\right)}$ and $x_{t}^{\left(2\right)}$ are analyzed. Co-integration finds coefficient *β* such that the linear combination of the two time series $y_{t}=x_{t}^{\left(1\right)}+\beta x_{t}^{\left(2\right)}$ is stationary.

Biologically, co-integrated population time series can result when the populations are regulated together but are not necessarily at an equilibrium point (e.g., they could be gradually recovering from past reduced abundance or declining due to over-exploitation). Co-integration can find associations among time series without identifying the source of a non-stationary pattern; this is advantageous in population time series analysis because such a pattern could be produced by many potential processes. Determining the source of non-stationary patterns using short time series is often very difficult. The co-integration method circumvents this problem. A more detailed description of the application of this method in population ecology is described in [23].

Because the power of co-integration analysis is weak [23], we applied it to one shrimp time series and one fish time series when both of them were non-stationary. The analysis was done using the Engle-Granger co-integration test [20]. An appropriate time lag for the residual regression for testing stationarity was chosen based on Akaike Information Criteria with a correction for finite data set (AIC_C_). For example, for the time lag of two, stationarity is tested using the following equation:

$$\Delta x_{t}=ax_{t-1}+b\Delta x_{t-1}+\varepsilon_{t}$$

where *a* and *b* are coefficients and *ε*_*t*_ is an independently identically distributed Gaussian error term. The time series *x*_*t*_ is considered stationary when *a* is not significantly different from 0. The stationarities of the original time series were tested by the Augmented Dickey-Fuller test using MATLAB function “adftest.m,” and the co-integration analysis was performed using MATLAB function “egcitest.m” [22].

Although co-integration analysis is a relatively new statistical method in population time series analysis, the method is well established in econometrics [20], and its application to population time series has been described [23]. The approach is conceptually similar to minimum/maximum autocorrelation factor analysis, which seeks a weighted linear combination of time series (factor) that maximizes a lag-one autocorrelation [24]. The factor, which has the smoothest pattern in the data, is interpreted as being produced by underlying population processes, and is thus analyzed further [25, 26]. However, such a pattern is found within dependent or independent variables without any information from the opposite variable. In contrast, co-integration finds the weighted linear combination of dependent and independent variables that removes a non-stationary pattern from the data. Populations exhibiting such time series are interpreted as being regulated together through external environmental factors and/or species interactions.

## Results

A total of 54 time series were analyzed (Figs 2 and 3). PLSR analysis was used to individually compare the six fish time series from each bay (three in each spring and fall) with two or three shrimp time series, for a total of 36 comparisons. The results revealed eight significant associations (Table 1), but there was no discernible pattern with respect to space or species.

**Fig 4:**
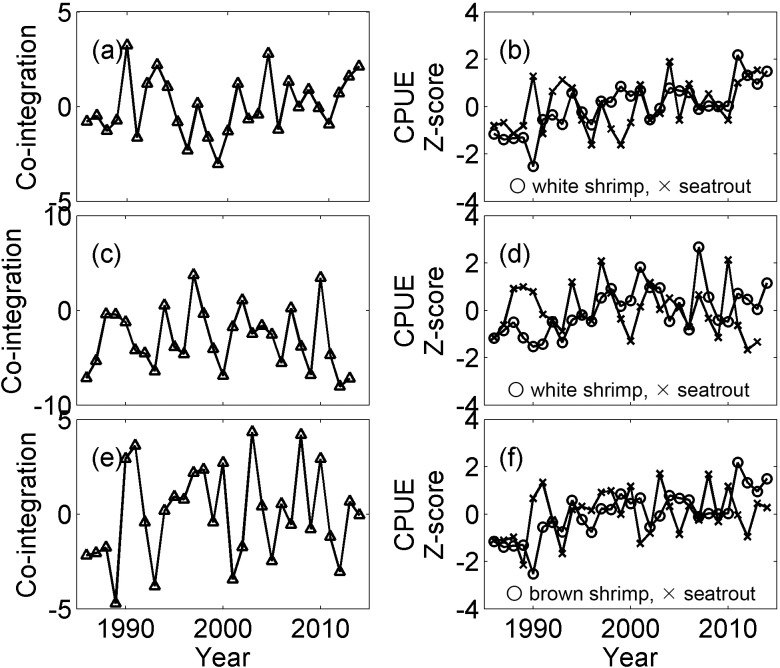
Co-integrated time series and catch per unit effort (CPUE) time series that are co-integrated in spring. Panels show: (a) co-integrated time series from Upper Laguna Madre; (b) white shrimp in August (circle) and spotted seatrout in spring (X) CPUEs in Upper Laguna Madre; (c) co-integrated time series from Galveston Bay; (d) white shrimp in November (circle) and spotted seatrout in spring (X) CPUEs in Galveston Bay; (e) co-integrated time series from Upper Laguna Madre; and (f) brown shrimp in May (circle) and spotted seatrout in spring (X) CPUEs. CPUEs were transformed by taking the square root and then standardized by taking the Z-score. The co-integrated time series shown in the left panels are the weighted linear combination of the time series shown in the right panels.

**Fig 5:**
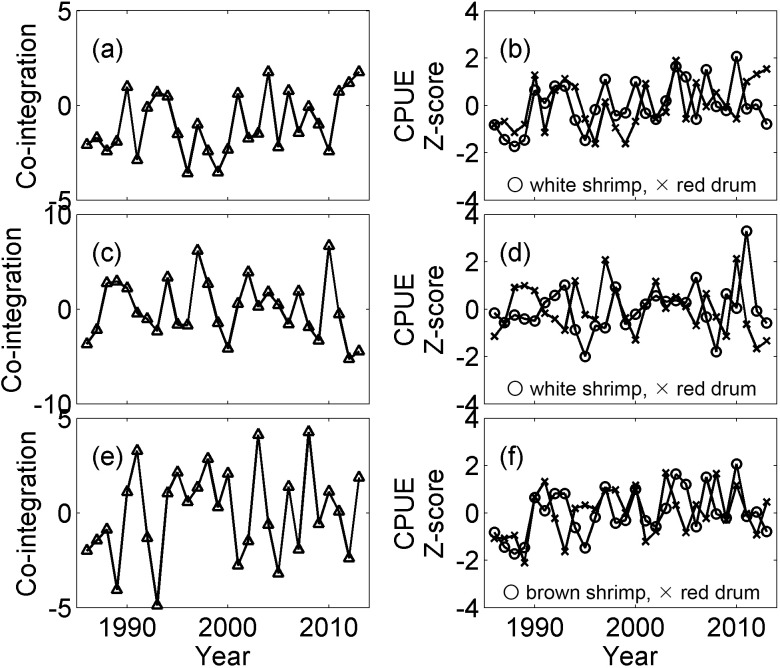
Co-integrated time series and catch per unit effort (CPUE) time series that are co-integrated in fall. The panels show: (a) co-integrated time series from Upper Laguna Madre; (b) white shrimp in August (circle) and red drum in fall (X) CPUEs in Upper Laguna Madre; (c) co-integrated time series from San Antonio Bay; (d) white shrimp in November (circle) and red drum in fall (X) CPUEs in San Antonio Bay; (e) co-integrated time series from Upper Laguna Madre; and (f) brown shrimp in May (circle) and red drum CPUEs in fall (X). CPUEs were transformed by taking the square root and then standardized by taking the Z-score. The co-integrated time series shown in the left panels are the weighted linear combinations of the time series shown in the right panels.

An Augmented Dickey-Fuller test suggested that a large number of the time series were non-stationary, but the lag-one differences of the time series were stationary. Because we were interested in finding non-stationary times series that become stationary through their linear combination (i.e., co-integrated time series), we eliminated the stationary time series from further analysis. In total, we compared 70 pairs (out of 108 possible pairs) of shrimp and fish time series using the Engle-Granger co-integration test. Under all of the comparisons, AIC_C_ suggested the time lag of two for residual regression. Of them, 31 were significant (Table 2). Inspection of these results further suggested that both white shrimp in August and brown shrimp in May were included in the associations for bays located on the lower coast of Texas, whereas white shrimp in November was more frequently found in the associations for the upper coast bay systems. All three species of fish investigated in this study appear to be affected by shrimp in all of regions. For each of the three shrimp time series, the fall and spring fish time series that exhibited the lowest *p*-values were selected and plotted in Figs 4 and 5.

**Table 2:** Results of the co-integration analysis. The *p*-values of pairwise comparisons are shown. Significant co-integration association (*p* ≤ 0.05) is indicated by bold and asterisk*. The lack of a *p*-value indicates that one or both original time series were stationary and no co-integration test was performed.

Location	Spring Fish Data			Fall Fish Data		
	Spotted Seatrout	Red Drum	Southern Flounder	Spotted Seatrout	Red Drum	Southern Flounder
Comparison with White Shrimp in August						
Galveston	0.23	0.15	0.22	0.17	0.20	0.15
Matagorda	--	--	--	--	--	--
San Antonio	0.36	0.42	0.43	0.41	0.41	0.43
Aransas	--	--	--	--	--	--
Corpus Christi	--	--	--	--	--	--
Upper Laguna Madre	0.00*	0.01*	0.01*	0.01*	0.00*	0.01*
Comparison with White Shrimp in November						
Galveston	0.00*	0.01*	0.00*	0.00*	0.00*	0.00*
Matagorda	0.02*	0.03*	0.03*	0.02*	0.03*	0.06
San Antonio	0.01*	0.00*	0.01*	0.01*	0.00*	0.00*
Aransas	--	--	--	--	--	--
Corpus Christi	--	--	--	--	--	--
Upper Laguna Madre	0.09	0.15	0.22	0.15	0.16	--
Comparison with Brown Shrimp in May						
Galveston	0.23	0.15	0.22	0.17	0.21	0.15
Matagorda	0.14	0.18	0.10	0.14	0.17	0.07
San Antonio	0.36	0.42	0.43	0.41	0.41	0.43
Aransas	0.02*	0.05*	0.08	0.05*	0.07	0.11
Corpus Christi	--	--	--	--	--	--
Upper Laguna Madre	0.00*	0.01*	0.01*	0.01*	0.00*	--

## Discussion

The objective of this study was to seek evidence supporting the importance of penaeid shrimp as a forage species in the Gulf of Mexico. We hypothesized that if the availability of two of the most abundant shrimp species in the region was vital for the sustainability of fish species, the fluctuations in prey and predator species abundance should have a statistical association. Although one should not conclude a trophic interaction based solely on a statistical association, we propose that such an association would support the existence of significant trophic interactions when combined with the knowledge that shrimp are commonly found in the stomachs of these fish species [13–17]. To this end, we tested for statistical associations between shrimp (brown and white) and three fish species (spotted seatrout, red drum, and southern flounder) in coastal bays of Texas using two statistical methods.

The results from our PLSR analysis suggested that there were some statistical associations between the shrimp and fish CPUEs, but we could find no discernible pattern with respect to space or species. Similar to linear regressions and associated analyses, the PLSR analysis is known to exhibit an inflated Type 1 error (false positive) when applied to non-stationary time series. As a large number of the time series in this study was non-stationary, the associations of 22% of the tested pairs (8 of 36 comparisons) could reflect such error. Conversely, time series data often suffer from large sampling errors, which can obscure the associations and increase Type 2 error (false negative). Therefore, we could not draw any conclusion based solely on the results from the PLSR analysis.

Unlike PLSR analysis, co-integration analysis is robust when used on non-stationary time series. Interestingly, our co-integration revealed that 31 of 70 pairs exhibited significant associations, and we could identify a discernible spatial pattern. Both white shrimp in August and brown shrimp in May were important in the bays of the south, whereas white shrimp in November was important in the bays of the north. This suggests that there may be a latitudinal influence. For example, temperature is known to influence the survival and growth of shrimp in the Gulf of Mexico [11, 12]. This was demonstrated with statistical modeling of field and laboratory experimental data [27] and field observation data [6], and laboratory experiments [28]. Therefore, it is possible that the latitudinal influence may be associated with a temperature gradient. However, salinity is also known to influence the vital rates of shrimp in the Gulf of Mexico [28–31], and freshwater input is higher in the northern bays than southern bays. Therefore, a salinity gradient is another possible environmental factor affecting shrimp abundance. Further studies on the influence of environmental factors on shrimp abundance are needed to understand the latitudinal gradient.

As co-integration identifies associations by finding a linear combination of data that produces a stationary time series, an identified association will not come from a year-to-year (i.e., high frequency) fluctuation. Instead, the association comes from a slowly changing (i.e. low frequency) pattern. Many potential causes of the low frequency pattern exist. For example, change in shrimping effort [32], deterioration of environmental conditions due to development [33] or climate change [34], or improvement of conditions due to restoration of estuaries [35] are possible causes.

On the other hand, high frequency fluctuations in shrimp CPUEs are likely from environmental fluctuations because both brown shrimp and white shrimp in the Gulf of Mexico exhibit weak stock-recruitment relationships [7, 36] and their total allowable catch in the region has not fluctuated with a high frequency, suggesting a strong influence of environmental conditions on their recruitment. However, the high frequency fluctuations are not translated into correlated high-frequency fluctuations in fish CPUEs because fish populations often include multiple ages. This process effectively acts as a moving average process, which is a low-pass filter, and the associations at high frequencies probably become statistical insignificant. It is unlikely that changes in the annual catch quotas of recreational fishing removed the associations at high frequencies because they would have also removed the associations at low frequencies.

As a part of our co-integration analysis, we also fitted a vector autoregressive (VAR) model with co-integrated variables to the data to generate a vector-error corrected (VEC) model [20]. The identification of any additional significant association would have suggested an association at a higher frequency. However, we found almost no further significant association. This result, which is consistent with those from our PLSR analysis, emphasized the lack of association among the time series with respect to a year-to-year (i.e., high frequency) fluctuation.

Behavioral differences between brown and white shrimp have been suggested to create differences in their vulnerability to potential predators. For example, brown shrimp were found to be more abundant in vegetated than non-vegetated areas, whereas white shrimp were less selective among habitat types [37]. This led to the hypothesis that white shrimp could be more vulnerable to predation than brown shrimp. However, our results suggest that the abundances of both shrimp species affect those of all three fish species with a regional (latitudinal) difference in their importance. This may be associated with their spatiotemporal distribution. The abundance of white shrimp in November (the sum of CPUEs from all years) was highest in Galveston Bay (the northernmost bay) and lower toward the south, whereas the abundances of white shrimp in August and brown shrimp in May were highest in San Antonio Bay, which is located approximately in the middle of the six bays. We hypothesize that the availability of shrimp relative to other potential prey species determines their importance as prey.

Commercial and/or recreational fishing for these species has a measurable impact on their populations and corresponding roles as prey or predators. Management actions have been required in response to shifts in population trends. By the mid 90’s the commercial shrimp fishery in Texas was overcapitalized and experiencing an excessive fishing effort, in order to address this situation, the Texas Parks and Wildlife Department introduced a limited entry and buyback program, which ended the sale of new commercial shrimp fishing licenses and provided funds for the purchase of existing licenses [38]. Through this program, over 65% of bay and bait licenses have been bought back and retired, resulting in a considerable reduction in shrimp fishing effort. While environmental variables also have an important role in shrimp recruitment, survival, and growth, past studies have shown that fishing effort alone accounts for 60 to 70% in shrimp catch variability [38]. Therefore, the observed low frequency fluctuations in shrimp abundance may be a result of the management actions.

Management actions have also affected populations of red drum, spotted seatrout, and southern flounder. Some of these actions include gear restrictions, such as a gill net ban in place since 1980 that benefited most fish species, or a more recent gigging ban in November, in response to a declining trend in southern flounder catch rates and designed to reduce fishing effort during their annual migration to the Gulf. Other actions include special designations, such as gamefish for red drum and spotted seatrout, which effectively ended the commercial fishery for these two species. Stock enhancement is another management measure that benefited red drum and spotted seatrout with about 25 million red drum and spotted seatrout juveniles produced and released every year into bay systems along the Texas’ coast in order to supplement their natural populations [39]. Finally, bag and size limits, adjusted through time depending on population trends, are also traditional management tools and applied to these species. Therefore, it is plausible that these independent management actions on the five species are obscuring or enhancing the statistical associations among the time series.

When the importance of a fishery stock is evaluated, the analysis often considers only the market value of the landed mass. However, our analysis supports the idea that penaeid shrimp in the Gulf of Mexico are also important as forage species for the three fish species that constitute an important resource for recreational fisheries in the region. These are the most sought after fish species in Texas, with spotted seatrout and red drum standing out as the two most frequently landed species in the coastal waters of Texas [10]. Thus, our findings suggest that we have underestimated the ecological value of shrimp stocks in the Gulf of Mexico. Moreover, because most of the shrimp biomass is produced in marshes and coastal wetlands [40], our findings stress the importance of protecting ecological integrity of such habitats with the goal of increasing the productivity of shrimp and the valuable fish species they support.

Any statistical analyses have associated potential errors, and co-integration analysis is not an exception. For example, an Augmented Dickey-Fuller test may spuriously categorize stationary time series as non-stationary. Then, a subsequent Engle-Granger co-integration test will conclude the existence of co-integration spuriously. However, in our analysis, the visual inspection of the original time series used in an Engle-Granger co-integration test suggests that many of them appear non-stationary. For example, the right panels of Figs 4 and 5 show the original time series used in the co-integration tests with highest p-values; those time series can be compared with the time series in Figs 2 and 3, which include both stationary and non-stationary time series used in the PLSR analysis. The Engle-Granger co-integration test is also sensitive to the choice of time-lag included. In this analysis, we used AIC_C_ to determine the time-lag because AIC_C_ selects the best model based on available data. However, any spurious conclusions with AIC_C_ will also lead to spurious results in the Engle-Granger co-integration. These two particular problems occur because of a general problem in statistical analysis where overall analysis is done in multiple steps. Consequently, statistical power and significance level for an overall statistical analysis are not the same as the statistical power and significance level of the final statistical analysis step (i.e., the Engle Granger co-integration test). We suggest this is one of the areas that still require further refinement with the method in the future.

## Conclusions

Here, by combining the results from PLSR and co-integration analysis, we conclude that the CPUE data suggest a significant association between shrimp and fish in the Gulf of Mexico. The abundances of shrimp and fish both exhibit low frequency patterns (i.e., non-stationary), and the association is found at this frequency. In contrast, year-to-year fluctuations do not necessarily show any significant association, likely because the responses of fish populations are not immediate (i.e., low inertia; [41]). This may have reduced the number of significant associations identified in our PLSR analysis. However, based on our results, we speculate that we could increase the number of significant associations identified in our PLSR analysis if we included an appropriate time lag and increased the length of the time series. Future research in this area could shed additional light on our conclusions.

## Supporting Information

S1 Table Original data used in the analysis. Catch per unit effort (CPUE) of fish data collected in fall, fish data collected in spring, white shrimp data collected in August, white shrimp data collected in November, and brown shrimp data collected in May.
(XLSX)
